# Use of electronic pharmacy transaction data and website development to assess antibiotic use in nursing homes

**DOI:** 10.1186/s12911-021-01509-7

**Published:** 2021-05-05

**Authors:** Sunah Song, Brigid M. Wilson, Joseph Marek, Robin L. P. Jump

**Affiliations:** 1 Cleveland Institute for Computational Biology, Cleveland, OH USA; 2 Department of Computer and Data Sciences, School of Engineering, Case Western Reserve University, Cleveland, OH USA; 3 Department of Population and Quantitative Health Sciences, School of Medicine, Case Western Reserve University, Cleveland, OH USA; 4 Division of Infectious Diseases and HIV Medicine in the Department of Medicine, School of Medicine, Case Western Reserve University, Cleveland, OH USA; 5 Geriatric Research Education and Clinical Center (GRECC), Veterans Affairs (VA) Northeast Ohio Healthcare System, 10701 East Blvd., Cleveland, OH 44106 USA; 6 CommuniCare Health Services, Cincinnati, OH USA

**Keywords:** Nursing home, Antimicrobial stewardship, Antibiotic stewardship, Fluoroquinolones

## Abstract

**Background:**

In 2017, the Centers for Medicare and Medicaid Services required all long-term care facilities, including nursing homes, to have an antibiotic stewardship program. Many nursing homes lack the resources, expertise, or infrastructure to track and analyze antibiotic use measures. Here, we demonstrate that pharmacy invoices are a viable source of data to track and report antibiotic use in nursing homes.

**Methods:**

The dispensing pharmacy working with several nursing homes in the same healthcare corporation provided pharmacy invoices from 2014 to 2016 as files formatted as comma separated values. We aggregated these files by aligning elements into a consistent set of variables and assessed the completeness of data from each nursing home over time. Data cleaning involved removing rows that did not describe systemic medications, de-duplication, consolidating prescription refills, and removing prescriptions for insulin and opioids, which are medications that were not administered at a regular dose or schedule. After merging this cleaned invoice data to nursing home census data including bed days of care and publicly available data characterizing bed allocation for each nursing home, we used the resulting database to describe several antibiotic use metrics and generated an interactive website to permit further analysis.

**Results:**

The resultant database permitted assessment of the following antibiotic use metrics: days of antibiotic therapy, length of antibiotic therapy, rate of antibiotic starts, and the antibiotic spectrum index. Further, we created a template for summarizing data within a facility and comparing across facilities. https://sunahsong.shinyapps.io/USNursingHomes/.

**Conclusions:**

Lack of resources and infrastructure contributes to challenges facing nursing homes as they develop antibiotic stewardship programs. Our experience with using pharmacy invoice data may serve as a useful approach for nursing homes to track and report antibiotic use.

**Supplementary Information:**

The online version contains supplementary material available at 10.1186/s12911-021-01509-7.

## Background

In the United States, nursing homes care for approximately 4 million individuals annually [1]. Antibiotics are some of the most frequently prescribed medications in nursing homes, with point prevalence studies indicating that on any given day, approximately 10% of nursing home residents receive antibiotics [2–5]. Unfortunately, 25–75% of antibiotic use in nursing homes is inappropriate [6–10]. Reasons to deem an antibiotic as inappropriate include an unnecessary indication (i.e., a viral infection), excessive length of therapy, selecting the wrong dose, or choosing an agent that is unnecessarily broad or does not treat the suspected bacterial pathogen. Systemic antibiotics may lead to adverse events including side effects, allergic reactions, drug-drug interactions, *Clostridioides difficile* infection (CDI), and selection for multi-drug resistant organisms (MDROs).

Antibiotic stewardship seeks to reduce inappropriate antibiotic use, which in turn supports patient safety and quality healthcare. Driven by the increasing prevalence of both CDI and MDROs across all healthcare settings [11, 12], the Centers for Medicare and Medicaid Services (CMS) required all nursing homes to have an antibiotic stewardship program by November 2017 [13]. In support of these efforts, the Centers of Disease Control and Prevention (CDC) developed a framework for antibiotic stewardship implementation called the Core Elements that detail actions critical for the success of antibiotic stewardship implementation [14]. Two of the seven Core Elements important to successful antibiotic stewardship programs are tracking and reporting antibiotic use. Many nursing homes lack resources, expertise, and infrastructure to assess antibiotic use in their buildings. Previous work has addressed the utility of using data from pharmacy invoices to measure antibiotic use in nursing homes [15]. Expanding on those efforts, we recently used pharmacy invoice data to characterize antibiotic use in 29 nursing homes in the United States [16]. Here, we describe in detail the process of transforming pharmacy invoices into a robust dataset that permits assessment of the following important antibiotic use metrics: overall days of therapy; length of therapy; antibiotic starts; use of intravenous agents; spectrum of antibiotic use based on specific antibiotic classes. We also developed a web interface that supports further analysis as well as tracking and reporting antibiotic use.

## Methods

### Data sources

The primary source of data was pharmacy invoices issued from a single dispensing pharmacy to several nursing homes within the same healthcare corporation. As previously described, invoice data accounts for medications requested by the nursing home for residents receiving skilled nursing care, for whom CMS is primary insurance provider [16]. We also obtained pharmacy dispensing records 6 nursing homes for data validation. These data indicate the medications dispensed from the pharmacy to the nursing home for residents receiving skilled nursing care (short-stay) and custodial or residential care (long-stay). The healthcare corporation provided monthly census data, indicating the bed-days of care, for individual nursing homes. Data describing nursing home characteristics came from the Long-Term Care: Facts on Care in the US (LTCFocus) websites and from Nursing Home Compare, both of which use data from CMS [17, 18]. The Institutional Review Board (IRB) at the VA Northeast Ohio Healthcare System approved the study protocol.

### Data aggregation

**Fig. 1:**
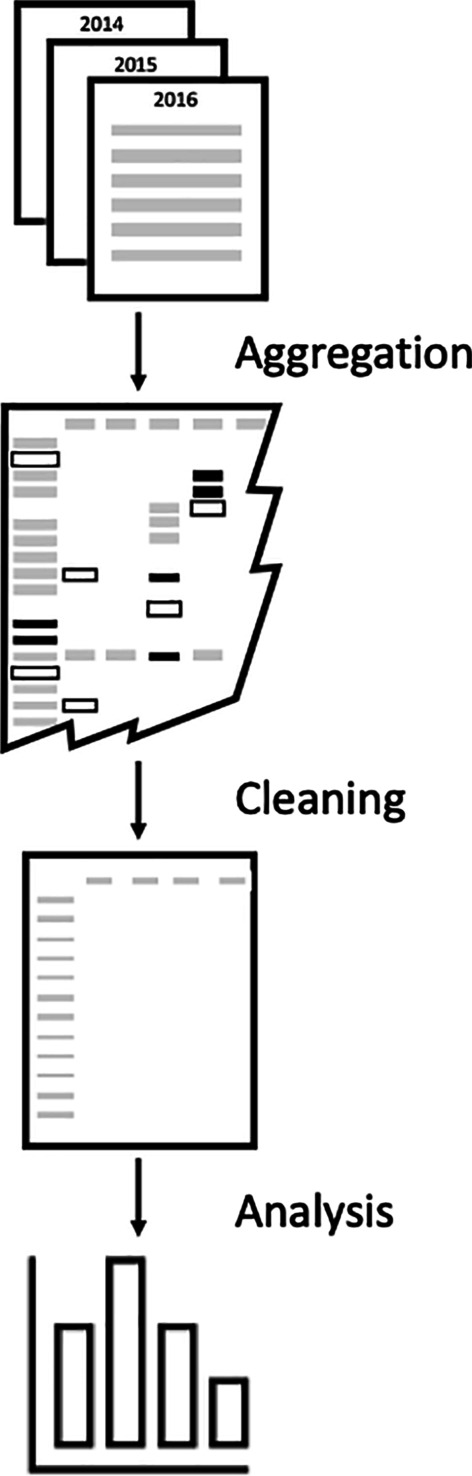
Conceptual overview of process used to transform pharmacy invoices into a database suitable for analysis. A single dispensing pharmacy provided several years of invoice data for nursing homes in the same healthcare corporation. We began with aggregating the data, which varied in the number and names of columns. This process revealed large gaps or irregularities in the data, such as a nursing home that entered or left the corporation during the study period; these nursing homes were removed. This yielded a single aggregated database with a consistent format. Data cleaning was a multi-step process leading to a dataset consisting of a single row to describe each prescription (including refills) for systemic medications administered on a regular schedule. This was the dataset used to analyze antibiotic use across a large number of nursing homes. Please see the text for further details

Transforming pharmacy invoices into a database suitable for analysis was a multistep process (Fig. 1). The healthcare corporation sent comma separated values (CSV) files containing invoices from January 2014 to June 2018. Each file included monthly invoices from several nursing homes, with each row in the file representing a transaction related to a prescription for an individual resident. We aggregated these files, which varied in the number of columns, into a single database with uniform column names and standardized data formats within each column. These data were then assessed for their distribution over time, by monthly intervals, as well as by individual nursing homes. Gaps or notable changes in the number of invoices from individual nursing homes were discussed with leadership at the healthcare corporation who provided context for some of these changes, such as a temporary closure or change in the resident population.

### Data cleaning

Data cleaning involved the following steps in sequence: removing rows with incomplete data, removing invoices for non-pharmaceutical items, removing lines indicating reimbursement, de-duplication, consolidating prescription refills into a single course, removing non-systemic medications, and removing insulin and opioids as administration of these medications often includes fluctuations in the dose or schedule. A clinician (RJ) and data scientist (BW) reviewed both the removed and retained data at each step to confirm it was clinically reasonable and accurate.

First, we removed rows that were missing any elements from the following essential columns: Facility Name, Transaction Date, Prescription Number, Drug Name, Days Supply, Amount, Route of Administration (i.e., oral or intravenous), Physician, and Health Care Payor. Second, we removed all rows pertaining to invoices for non-pharmaceutical agents (e.g., kits for placing intravenous lines or normal saline). Third, rows for reimbursement or payment, indicated by negative monetary values, were removed. Fourth, we reconciled records for which we found multiple rows with the same prescription number, medication, and transaction date according to the logic below for the two scenarios observed:

- *Case One* If there was more than one row with the same prescription number, medication, transaction date, physician, and days supply, then we retained only one row.
- *Case Two* If there was more than one row with the same prescription number, medication, transaction date, physician and different days supply, we retained the row with the greatest number of days supply.

Fifth, we addressed instances of more than one row with the same prescription number and medication but with different transaction dates, with later transaction dates representing medication dispensing events or refills. To consolidate multiple rows with the same prescription number, we added up the total days supply across rows and considered this to be the length of therapy. Sixth, we used several regular expressions to search for and remove rows for non-systemic medications, *i.e.*, medications administered topically, as otic or ophthalmologic drops, as suppositories, or via inhalation (Table 1). This left rows for medications administered through oral, intravenous, and intramuscular routes. Finally, we used regular expressions specific to insulin and opioids to remove rows for these medications, which are frequently administered using a variable dose and schedule.

**Table 1:** Regular expressions used to remove non-systemic medications

Regular expressions	Description and example	Strings
[^[:alpha:]]STRING	Drug Name that ends with [Non Alphabetic Character]STRING e.g.*,* HYDROCORTISONE 2.5% CREAM	CREAM, CRM, DROP, EAR, EYE, EYEOINT, FOAM, GEL, INHALER, INHAL, LOTION, MOUTHWAS, MOUTHWASH, NASAL, OINT, OINTM, OINTMENT, OPTH, OPHTH, OTIC, PATCH, RINSE, SPRAY, TOPIC, TOPICAL, SHAMP, SHAMPOO, RINGERS, 1:1
[^[:alpha:]]STRING[^[:alpha:]]	Drug Name contains [Non Alphabetic Character]STRING[Non Alphabetic Character] e.g.*,* PERMETHRIN CREAM 5% 60GM	CREAM, CRM, DROP, EAR, EYE, EYEOINT, FOAM, GEL, INHALER, INHAL, LOTION, MOUTHWAS, MOUTHWASH, NASAL, OINT, OINTM, OINTMENT, OPTH, OPHTH, OTIC, PATCH, RINSE, SPRAY, TOPIC, TOPICAL, SHAMP, SHAMPOO, RINGERS, 1:1
^STRING[^[:alpha:]]	Drug Name that starts with STRING[Non Alphabetic Character] e.g.*,* GELFOAM SIZE 50 SPONGE	GELFOAM, WATER INJ, SSD, ABH
^STRING[^[:blank:]]%*	Drug Name that starts with STRING[blank] and zero or more times of % e.g.*,* LIDOCAINE HCL 2% JEL	DS, LIDOCAINE, NACL, SODIUM CHL

### Data analysis

From the resulting set of systemic medications, we identified antibiotics, categorizing these into several subclasses, as well as anti-hypertensives from several subclasses (beta-blockers, angiotensin-converting enzyme inhibitors [ACE-Is] and angiotensin 2 receptor blockers [ARBs]) (Additional file 1: Table S1). The rational for using anti-hypertensives is that these agents are commonly prescribed, including among nursing home residents, and have not been subject to recent changes in guidance or recommendations that might lead to changes in their use. In combination with census data, we quantified systemic medications by the number of starts per 1000 bed days of care (BDOC), days of therapy (DOT) per 1000 BDOC, and length of therapy (in days). Both the number of starts/1000 BDOC and DOT/1000 BDOC are common metrics to assess antibiotic use [19, 20]. The number of starts/1000 BDOC assesses the frequency of prescribing. DOT/1000 BDOC is the number of calendar days a medication is administered standardized to total days in care and describes the overall rate of use; we determined this based on the days supply [21, 22]. For some medications, particularly those with several refills or with a transaction date near the end of a month, the total DOT exceeded the calendar days in that month. Accordingly, for calculating DOT/1000 BDOC at monthly intervals, we distributed the DOT across months. For example, if an anti-hypertensive was prescribed for 90 days starting December 3, 2014, the DOT would be allocated as follows: 28 DOT for December 2014, 31 DOT for January 2015, 28 DOT for February 2015 and 3 DOT for March 2015 for a total of 90 DOT.

We also used previously described antibiotic spectrum index (ASI) to summarize prescribed antibiotics on a quantitative scale from 1 to 13, with lower values indicating more narrow-spectrum agents and higher values more broad-spectrum agents (Additional file 1: Table S2) [23]. We calculated the monthly mean ASI among the antibiotic DOT for each facility.

### Data validation

To validate our analysis dataset, we used dispensing data from 2016 for six of the nursing homes. These data were cleaned and analyzed using the same process described for the larger invoice dataset. We used the Mann–Kendall trend test and Spearman rank correlation test to compare antibiotic DOT/1000 BDOC from the invoice and dispensing datasets.

### Website development

Using an open source Shiny R package, we built an interactive web application to show the result of each steps for data cleaning and further antibiotic use analysis [24]. The shinyapps.io cloud hosting allowed us to deploy the application online in secure environment. The application runs in its own protected domain on the cloud server and user access is always secure sockets layer (SSL) encrypted.

## Results

### Data aggregation

The dispensing pharmacy provided a total of 51 files with 15 to 27 columns describing transactions from 1/1/2014 through 6/30/2018. We used column headers and nursing homes names to reconcile the meta-data, generating a file with 28 columns and nearly 2 million rows including 82 distinct nursing home names. Only 11 columns were retained for the aggregation; of the discarded columns, 11 of the columns had > 40% missing data and six additional columns had > 90% missing data (Table 2).

**Table 2:** Column headings used to aggregate files

Final column name	Original Column Name(s)	Description of data elements
Facility name	Facility name	82 distinct expressions for names of nursing homes
Medication	Drug name, medication name, agent, description	13,549 distinct medications
Dose	Dose, quantity, qty	Quantities in grams or milligrams for oral medications and in gm/mL for intravenous medications
Days supply	Days supply, days of supply, days dispensed	Ranged from 0 to 946 days
Prescription number	Prescription number, Rx	938,007 distinct numbers
Transaction date	Transaction date	Ranged from 9/23/2007 to 6/25/2018
Amount	Amount	Ranged from $0 to $15,000.08
Route of administration	Route of administration, inventory category	40 distinct expressions
Physician	Physician	7971 distinct physicians
Health care payor	Payor, pay type description	13 distinct descriptions reflecting the entity, typically an insurance company, that ultimately pays for the medication, listed as general categories of Medicare, Medicaid, the nursing home, Veterans Affairs, hospice, and private parties
National drug code	NDC	National Drug Code, 7% missingness, 9885 distinct codes. Ultimately, not used in data analysis

Among the 82 distinct nursing home names, we excluded 37 nursing home names that contributed ≤ 12 months of data to the study period (1/1/2014–12/31/2016). We reviewed the remaining 45 nursing homes names and noted that some were very similar. In consultation with leadership from the healthcare corporation, we consolidated similar names. For example, we assigned ‘SleepyHollow’ as a facility name for rows that contained ‘SleepyHollow’, ‘Sleepy Hollow’, or ‘Sleep Hollow CCRC’; CCRC is an acronym for continuing care retirement community. This resulted in 32 distinct nursing home names.

For each nursing home, we assessed the number of rows attributed to each month from 2014 to 2016. We found that three nursing homes had a > 20% change in the number of rows over three consecutive months. Discussion with leadership from the healthcare corporation revealed that one building had closed and that two had changed the population served sufficiently to alter the volume of pharmacy invoices (i.e., from those in need of skilled nursing care to those with psychiatric illness). Removing these facilities from the list resulted in a database with ten essential columns and 995,785 rows across 29 nursing homes.

### Data cleaning

The primary aim of data cleaning was to create a database with one row per prescription that included complete data including the prescribed drug, the date of the prescription, the duration of the prescription, the location (nursing home) of the prescription, and the prescribing provider. To achieve this aim, we took the following steps that generally consisted of excluding, aggregating, or reconciling rows within our starting database.

From the aggregated database, only 18,525 (1.8%) of rows had data missing from the ten columns deemed essential for analysis; these were removed (Table 3). Rows describing non-pharmaceutical items and records indicating reimbursement were also removed, leaving 648,326 rows. When we considered the two different cases of duplications, Case One (same prescription number, medication, transaction date, physician, and days supply) accounted for 48,088 rows removed and Case Two (same prescription number, medication, transaction date, physician, and different days supply) accounted for 10,663 rows removed.

**Table 3:** Rows removed when cleaning aggregated dataset

Step	Description	Rows removed	Rows remaining (%)
(n/a)	Records in the aggregated dataset		995,785
1	Removed rows missing data from essential columns	18,525	977,260 (98%)
2	Removed rows for non-pharmaceuticals	99,362	877,898 (88%)
3	Removed rows indicating reimbursements	229,572	648,326 (65%)
4	Removed duplicate rows	58,751	589,575 (59%)
5	Removed rows indicating medication refills	136,172	453,403 (46%)
6	Removed rows for non-systemic medications	35,899	417,504 (42%)
7	Removed rows for insulin and opioids	49,208	368,296 (37%)

Of the remaining 589,575 lines of data, there were 136,172 rows that did not have a unique prescription number. Rows with the same prescription number indicated a dispensing event from the pharmacy of the same medication at the same dose to the same person. Most often, these were refills for medications used to treat chronic conditions, such as anti-hypertensives for high blood pressure. In other instances, these represented several dispensing events for a limited number of doses of a medication with a limited shelf-life, most often intravenous antibiotics. For both situations, we added up the total days supply among rows with the same prescription number.

In the final step of data cleaning, we used regular expressions to remove rows for non-systemic medications (35,899 rows) and rows for medications administered without a fixed dose or schedule, specifically insulin and opioids (49,208 rows). The clean database had 368,296 rows, which was 37% of the those present in the aggregated dataset.

As previously described, we used the clean database to describe antibiotic use across 29 US nursing homes [16], which included steps that allowed us to summarize antibiotic use at yearly intervals based on the date and duration of a prescription. Allocating the DOT across calendar months indicated that 54% of prescriptions carried over to more than 1 month and 6% extended for more 2 months or longer. Further analysis was performed after merging nursing home census data (e.g., bed days of care, occupancy rate) to the cleaned antibiotic use data to standardize antibiotic use to bed days of care and analyze as rates. We further merged data elements used to describe operational characteristics, resident demographics, and quality indicators specific to nursing homes [17, 18]. In summary, our results indicated the following: between 2014 and 2016, the overall rate of antibiotic use did not change; fluoroquinolones were the most commonly prescribed class of antibiotics; and the mean ASI was similar across nursing homes [16]. When assessed based on the number of beds, we did not observe notable differences in rates of total antibiotic use among nursing homes with < 100 beds compared to those with 100–150 and those with > 150 beds.

### Data validation

We validated the antibiotic use data derived from invoice data with dispensing data obtained for six nursing homes. Antibiotic DOT/1000 BDOC based on dispensing data was consistently greater than that those based on invoice data (Additional file 1: Figure S1). This is consistent with the resident populations encompassed by these datasets: dispensing data accounts for all residents in the nursing home while invoice data accounts primarily for residents receiving skilled nursing care. Comparing the monthly 2016 measures from both data sources, the Spearman rho correlation for antibiotic DOT/1000 BDOC was high (> 0.7) for two nursing homes and moderate (0.5–0.7) for three nursing homes. When we focused on intravenous antibiotics, typically administered only to residents receiving skilled nursing care and thus expected to occur in invoice data with a CMS payor, the Spearman rho correlation values increased for five of the six nursing homes. For one of the nursing homes, the correlation decreased from 0.81 to 0.39. Leadership from the healthcare corporation confirmed that the operational characteristics for this nursing home were similar to the others and suggested the possibility of errors related to transfer of data specific to compounded intravenous medications, which are often handled through a different software system.

### Website

**Fig. 2:**
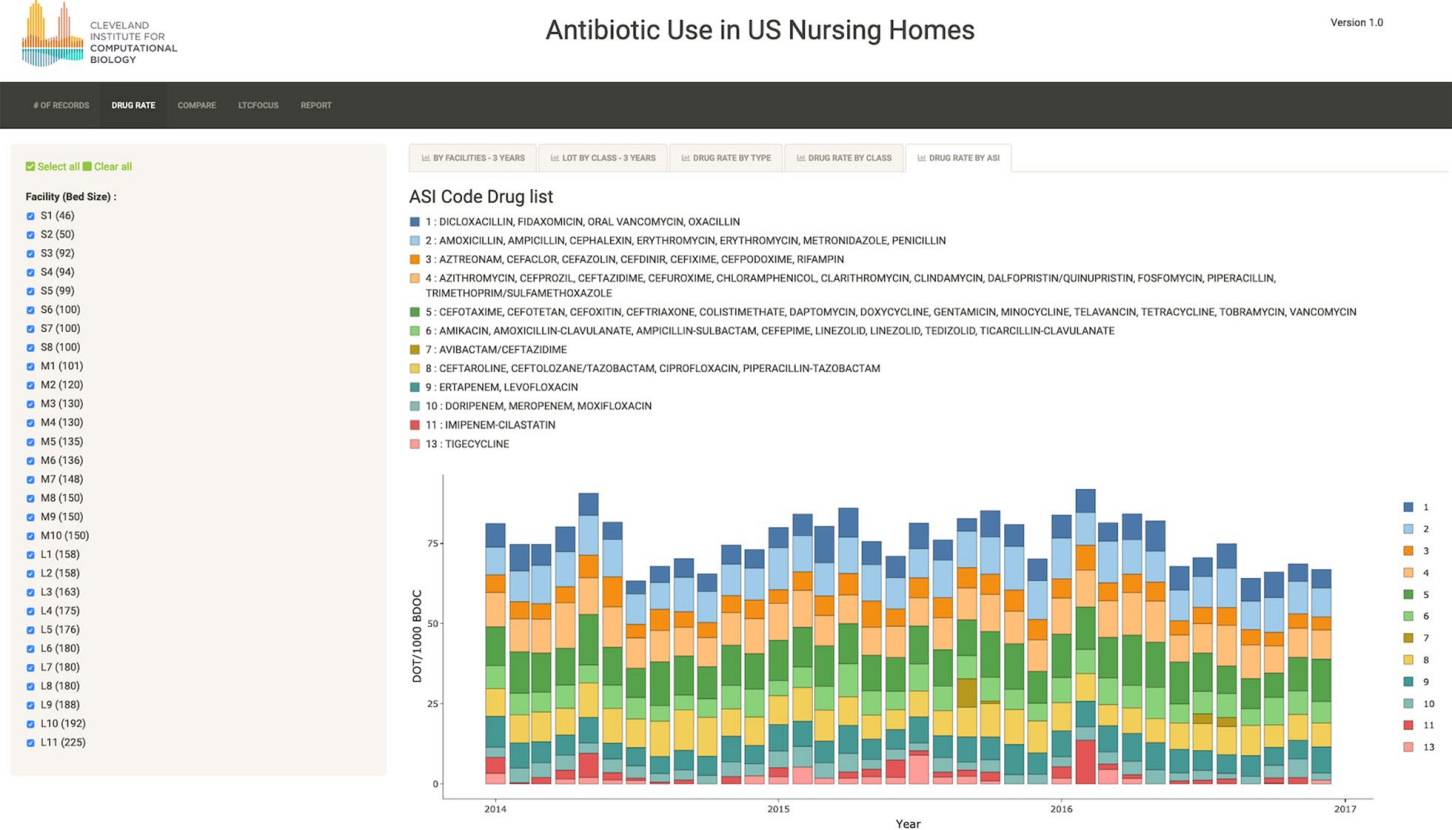
The website offers a comprehensive and detailed interface. Users may easily parse data for specific nursing homes by year and/or month and compare results among individual or groups of nursing homes. The website also allows users to assess antibiotic use relative to an array of nursing home characteristics. Finally, the website permits users to generate an antibiotic use report that compares their nursing homes against others, with data elements normalized to bed days of care when appropriate. The graph shows the antibiotic spectrum index (ASI) for all nursing homes studied from 2014 to 2016, stratified by antibiotic days of therapy per 1000 bed days of care

**Fig. 3:**
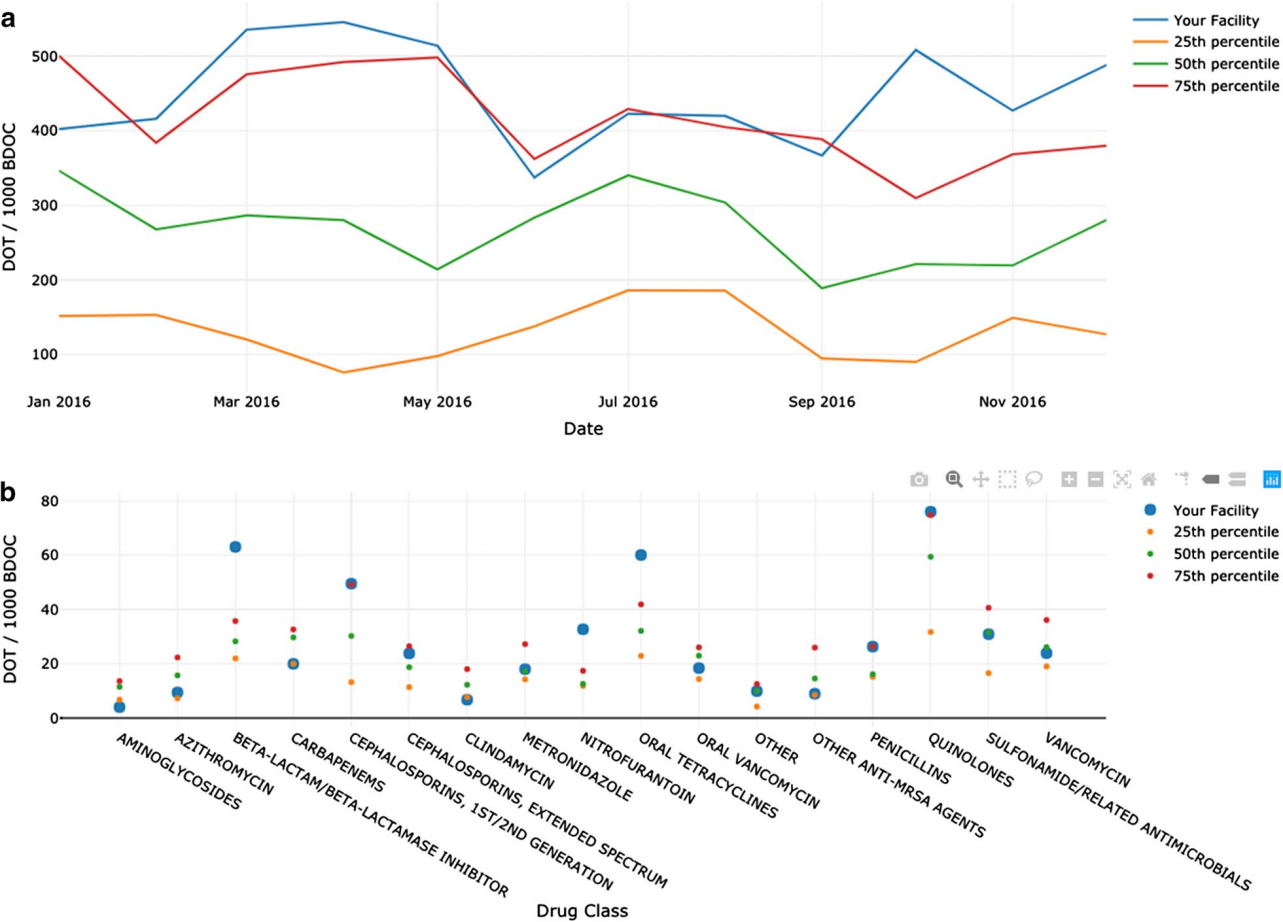
Comparative feedback (or benchmarking) report comparing antibiotic use at one nursing home to others of a similar size. **a** Overall antibiotic use as Days of Therapy per 1000 Bed Days of Care (DOT/1000 BDOC). **b** Rates of use for specific antibiotic classes and agents. Note the tools in the upper right aspect of the graph that are available to support more nuanced visualization of the data

Figure 2 shows the interface, which included an array information organized into five tabs. The first tab, Number of Records, provided an overview of the number of records removed with each cleaning step. The second tab, Drug Rates, had five sub-tabs that gave access to several antibiotic use metrics including overall rates of antibiotic use, length of antibiotic therapy, antibiotic use categorized by class of agent, and the antibiotic spectrum index (shown in the graph in Fig. 2), which provides a quantitative comparison of the activity of antibiotics against bacterial pathogens. An additional sub-tab compared antibiotic use to all systemic medications and to use of anti-hypertensive agents. With the third tab, Compare, users could parse nursing homes into two subgroups and use robust data visualization to compare several antibiotic use metrics. The fourth tab, LTCFocus, provided data and graphs that describe relationships between antibiotic use and nursing home characteristics, as assessed by CMS and made available through LTCFocus.org [17]. Finally, the Report tab generated two simple graphs that provide comparative feedback so that both clinical and administrative staff can understand how antibiotic use at their facility compares to antibiotic use at nursing homes with a similar number of beds (Fig. 3). Filters available within the website permitted users to examine individual or groups of nursing homes and to specify the time frame assessed. Another feature, labeled Data Table and at the bottom on most of the tabs, permitted users to download data for further analysis. Across all the tabs, tooltips are also available when the user hovers over, focuses on, or touches an element of figures. Additional details of results are available in the shiny repository, https://sunahsong.shinyapps.io/USNursingHomes/.

## Discussion

Our intent was to provide a blueprint to support nursing homes in using an existing dataset, pharmacy invoices, to construct a dataset that permits tracking and reporting antibiotic use. Analyzing the data derived from the cleaning approach described here, we observed a frequency of fluoroquinolone use as well as steady rates of antibiotic use between 2014 and 2016 that were consistent with other reports that use clinical or surveillance datasets to assess antibiotic use in US nursing homes [5, 15, 25, 26]. Validation of a subset of the data derived from invoice data using pharmacy dispensing data found similar overall trends for total antibiotic use and for intravenous antibiotic use in five of the six nursing homes assessed. These coupled results support the use of cleaned invoice data as a valid measure of antibiotic use. 4/28/2021 4:23:00 PMFurthermore, we developed a website specifically designed to serve as an interface that ready visualization of several antibiotic use metrics. The approach described and the resulting website support comparing antibiotic use among nursing homes within a network or region served by the same dispensing pharmacy.

The strengths of the website are the displays that permit visualization of changes over time as well as comparisons to other sites. The user-friendly design encourages changing views to answer questions specific to an individual site. Furthermore, while tracking and reporting antibiotic use typically falls under the purview of a nursing home’s infection preventionist(s), the members of the antibiotic stewardship team or the Quality Assurance and Performance Improvement (QAPI) committee could also use the website. They may be interested in assessing changes following an intervention, such as restricting use of fluoroquinolones, the most commonly prescribed antibiotic class, to residents with severe penicillin allergies. Examination of antibiotic use over time makes it possible to detect unintended consequences of changes. Building on the previous example, restricting use of fluroquinolones may lead to an increase in other antibiotics, such as clindamycin or doxycycline. Inclusion of identifying information may enable assessment of additional antibiotic stewardship metrics, such as conversion from intravenous to oral antibiotic administration.

While the steps of data aggregation and cleaning described above are applicable to invoice formats present in the administration of most nursing homes, the expertise needed to transform pharmacy invoice data into a clean database, and to maintain those processes, may be available only to nursing homes that are part of a larger healthcare system. Guidelines for hospital-based antibiotic stewardship programs call for a core multidisciplinary antimicrobial stewardship team that includes an infectious diseases-trained physician and clinical pharmacist as well as a clinical microbiologist, hospital epidemiologist, information specialist, and an infection preventionist [27]. Except for an infection preventionist, individual nursing homes do not typically employ individuals with these skill sets. Indeed, CMS indicates that in nursing homes, the antibiotic stewardship program is part of the broader infection prevention and control program, which is led by one or more infection preventionists [13]. CMS estimates that for the average nursing home, only 15% of a full-time equivalent (FTE) is required for the entirety of the infection prevention and control program [13]. The disproportionate effect of the COVID-19 pandemic on nursing home residents, which account for only 4% of cases and over 33% of deaths in the US [28], is a strong indicator more resources are needed to support infection prevention and control in nursing homes, which in turn will enhance resident safety. We suggest that 15% of an FTE may be an adequate allocation specifically for tracking and reporting antibiotic use in nursing homes. Additional effort from a clinical pharmacist or physician may be necessary to provide oversight and help with data interpretation.

Most hospitals use information from their electronic medical records to assess antibiotic use; this can include medication orders, dispensing events, and bar-coded medication administration (BCMA) data. Previous work at a Veterans Affairs hospital compared medical orders with BCMA data to assess antibiotic metrics. While both approaches had errors, BCMA data was more accurate, consistent with this being a process that confirms that the correct medication at the correct dose is being given to the correct patient at the point of care [29]. As assessment of BCMA data from 13 nursing homes, however, found an error rate of 90%, with most errors related to the timing of medications that require administration (i.e., every 4 to 6 h) [30]. While these findings belie the expectation of strong medication adherence in all institutionalized settings, using electronic medical record to assess antibiotic use in nursing homes would likely be superior to pharmacy invoice data [31]. Not all nursing homes have adopted electronic medical records and those that have may not have personnel with time and expertise to extract antibiotic use data for tracking and reporting purposes.

Invoice data serves as a proxy for antibiotic use in nursing homes, with limitations that include not capturing changes from intravenous to oral administration or accounting for discontinuation of antibiotics. Kabbani et al*.* also used invoice data to assess antibiotic use. In contrast to the dataset discussed here, their invoices did not have the prescription numbers that permitted us to link dispensing events related to a single medication. Even with this limitation, Kabbani et al*.* [15] were able to compare the antibiotic days of therapy among 12 nursing homes, finding a five-fold difference in rates between the lowest- and highest-use homes. The use of dispensing data would likely permit more accurate assessment of discontinued medications, which would help to improve the accuracy for antibiotic use metrics in intravenous agents in particular, as these may requiring multiple dispensing events for a single course. Dispensing data, however, may not be as readily accessed as invoice data.

Our approach has limitations. First, the website is designed to permit comparison of several affiliated nursing homes that are served by a single dispensing pharmacy. Independent nursing homes may still use the process outlined, along with the website, to assess antibiotic use over time. Second, the steps used to clean the data may not apply to data format used by some pharmacy invoice systems. We assert that the general approach outlined will serve as a template for information technologists to work directly with their own data. They may be able to use additional data fields, such as the indications for antibiotics prescribed, to yield further information relevant to antibiotic stewardship efforts. Third, like any system, errors may accrue. Minor discrepancies inherent to using pharmacy invoices include unbilled single-dose medication administration, cancelled orders, changes in treatment, or resident transfers. As long as these events remain at a reasonably low and fairly consistent level, they should not interfere with the overall meaning of antibiotic use data. More notable problems with the data are likely be noticed by end-users who will need to work with information technologists to identify potential sources of error and validate the data.

## Conclusions

The process for transforming pharmacy invoices into antibiotic use data may help support overall antibiotic stewardship efforts for some nursing homes. Readily tracking and reporting antibiotic use should complement other efforts, such as those of nursing homes participating in the National Healthcare Safety Network (NHSN), which supports tracking urinary tract infections, *C. difficile* infections and multidrug-resistant organisms [32]. Ultimately, our hope is that this approach will reduce some of the barriers to tracking and reporting antibiotic use and will become another tool used by nursing homes to improve the use of antibiotics and thus, enhance the safety and quality of care for their residents.

## Supplementary Information

**Additional file 1**: **Table S1.** Lists the class, subclass, and name of the antibiotics and anti-hypertensive agents evaluated. Table S2 details the Antibiotic Spectrum Index scores for individual antibiotics. **Figure S1.** Compares antibiotic use for six nursing homes based on data derived the pharmacy invoices and from dispensing data.

## Data Availability

The datasets generated during the current study are available in the shiny repository, https://sunahsong.shinyapps.io/USNursingHomes/.

## Abbreviations

GRECC: Geriatric Research Education and Clinical Center
VA: Veterans Affairs
CDI: *Clostridioides difficile* Infection
MDRO: Multi-drug resistant organism
CMS: Centers for Medicare and Medicaid Services
CDC: Centers of Disease Control and Prevention
LTCFocus: Long-Term Care: Facts on Care in the US
CSV: Comma separated values
ACE-I: Angiotensin-converting enzyme inhibitor
ARB: Angiotensin 2 receptor blocker
DOT: Days of therapy
BDOC: Bed days of care
ASI: Antibiotic spectrum index
CCRC: Continuing care retirement community
US: United States
NHSN: National Healthcare Safety Network
IRB: Institutional Review Board
