# Supplementary material for: Use of electronic pharmacy transaction data and website development to assess antibiotic use in nursing homes
Source: BMC Med Inform Decis Mak. 2021 May 5;21:148. doi: 10.1186/s12911-021-01509-7 (PMC8097250; doi:10.1186/s12911-021-01509-7)
Supplement: Supplementary file 1 — Additional file 1: Table S1. Lists the class, subclass, and name of the antibiotics and anti-hypertensive agents evaluated. Table S2 details the Antibiotic Spectrum Index scores for individual antibiotics. Figure S1. Compares antibiotic use for six nursing homes based on data derived the pharmacy invoices and from dispensing data. [file 12911_2021_1509_MOESM1_ESM.docx]

Additional File 1

**Table S1. Medications by Class and Subclass**

| **Class** | **Subclass** | **Medication** |
| --- | --- | --- |
| Antibiotic | AMINOGLYCOSIDES | AMIKACIN |
|  |  | GENTAMICIN |
|  |  | TOBRAMYCIN |
| Antibiotic | AZITHROMYCIN | AZITHROMYCIN |
| Antibiotic | BETA-LACTAM/BETA-LACTAMASE INHIBITOR | AMOXICILLIN/CLAVULANATE |
|  |  | AMPICILLIN/SULBACTAM |
|  |  | AVIBACTAM/CEFTAZIDIME |
|  |  | CEFTOLOZANE/TAZOBACTAM |
|  |  | PIPERACILLIN/TAZOBACTAM |
| Antibiotic | CARBAPENEMS | CILASTATIN/IMIPENEM |
|  |  | DORIPENEM |
|  |  | ERTAPENEM |
|  |  | MEROPENEM |
| Antibiotic | CEPHALOSPORINS, 1ST/2ND GENERATION | CEFACLOR |
|  |  | CEFADROXIL |
|  |  | CEFAZOLIN |
|  |  | CEFOTETAN |
|  |  | CEFOXITIN |
|  |  | CEFPROZIL |
|  |  | CEFURIXOIME |
|  |  | CEPHALEXIN |
| Antibiotic | CEPHALOSPORINS, EXTENDED SPECTRUM | AZTREONAM |
|  |  | CEFDINIR |
|  |  | CEFEPIME |
|  |  | CEFIXIME |
|  |  | CEFOTAXIME |
|  |  | CEFPODOXIME |
|  |  | CEFTAROLINE |
|  |  | CEFTAZIDIME |
|  |  | CEFTRIAXONE |
| Antibiotic | CLINDAMYCIN | CLINDAMYCIN |
| Antibiotic | METRONIDAZOLE | METRONIDAZOLE |
| Antibiotic | NITROFURANTOIN | NITROFURANTOIN |
|  | ORAL TETRACYCLINES | DOXYCYCLINE |
|  |  | MINOCYCLINE |
|  |  | TETRACYCLINE |
| Antibiotic | ORAL VANCOMYCIN | ORAL VANCOMYCIN |
| Antibiotic | OTHER | CLARITHROMYCIN |
|  |  | COLISTIMETHATE |
|  |  | DALFOPRISTIN/QUINUPRISTIN |
|  |  | ERYTHROMYCIN |
|  |  | FIDAXOMICIN |
|  |  | FOSFOMYCIN |
|  |  | TEDIZOLID |
|  |  | TELAVANCIN |
|  |  | TIGECYCLINE |
| Antibiotic | OTHER ANTI-MRSA* AGENTS | DAPTOMYCIN |
|  |  | LINEZOLID |
| Antibiotic | PENICILLINS | AMOXICILLIN |
|  |  | AMPICILLIN |
|  |  | DICLOXACILLIN |
|  |  | NAFCILLIN |
|  |  | OXACILLIN |
|  |  | PENICILLIN |
| Antibiotic | QUINOLONES | CIPROFLOXACIN |
|  |  | LEVOFLOXACIN |
|  |  | MOXIFLOXACIN |
| Antibiotic | SULFONAMIDE/RELATED ANTIMICROBIALS | BACTRIM |
|  |  | SULFAMETHOXAZOLE/TRIMETHOPRIM |
|  |  | TRIMETHOPRIM |
| Antibiotic | VANCOMYCIN (intravenous) | VANCOMYCIN |
| Anti-hypertensive | Beta-Blockers | ACEBUTOLOL  ATENOLOL  ATENOLOL-CHLORTHALIDONE  BISOPROLOL  BISOPROLOL-HYDROCHLOROTHIAZIDE  BYSTOLIC  CARVEDILOL  COREG  LABETALOL  METOPROLOL  METOPROLOL-HYDROCHLOROTHIAZIDE  NADOLOL  PINDOLOL  PROPRANOLOL  SOTALOL  TIMOLOL |
| Anti-hypertensive | Angiotensin Converting Enzyme Inhibitors (ACE-I) | AMLODIPINE-BENAZEPRIL  BENAZEPRIL  BENAZEPRIL-HYDROCHLOROTHIAZIDE  CAPTOPRIL  ENALAPRIL  ENALAPRIL-HYDROCHLOROTHIAZIDE  FOSINOPRIL  LISINOPRIL  LISINOPRIL-HYDROCHLOROTHIAZIDE  MOEXIPRIL  MOEXIPRIL-HYDROCHLOROTHIAZIDE  QUINAPRIL  QUINAPRIL-HYDROCHLOROTHIAZIDE  RAMIPRIL  ZESTRIL |
| Anti-hypertensive | Angiotensin 2 Receptor Blockers (ARBs) | AMLODIPINE-VALSARTAN  AMLODIPINE-VALSARTAN-HYDROCHLOROTHIAZIDE  AVAPRO  BENICAR  CANDESARTAN  CANDESARTAN-HYDROCHLOROTHIAZIDE  DIOVAN  ENTRESTO  IRBESARTAN  IRBESARTAN-HYDROCHLOROTHIAZIDE  LOSARTAN  LOSARTAN-HYDROCHLOROTHIAZIDE  TELMISARTAN  TELMISARTAN-HYDROCHLOROTHIAZIDE  VALSARTAN  VALSARTAN-HYDROCHLOROTHIAZIDE |
|  |  |  |

*MRSA, methicillin-resistant *Staphylococcus aureus*

**Table S2.** Drug list for Antibiotic Spectrum Index (ASI)^a^

| **ASI** | **Drugs** |
| --- | --- |
| 1 | DICLOXACILLIN, FIDAXOMICIN, ORAL VANCOMYCIN, OXACILLIN |
| 2 | AMOXICILLIN, AMPICILLIN, CEPHALEXIN, ERYTHROMYCIN, ERYTHROMYCIN, METRONIDAZOLE, PENICILLIN |
| 3 | AZTREONAM, CEFACLOR, CEFAZOLIN, CEFDINIR, CEFIXIME, CEFPODOXIME, RIFAMPIN |
| 4 | AZITHROMYCIN, CEFPROZIL, CEFTAZIDIME, CEFUROXIME, CHLORAMPHENICOL, CLARITHROMYCIN, CLINDAMYCIN, DALFOPRISTIN/QUINUPRISTIN, FOSFOMYCIN, PIPERACILLIN, TRIMETHOPRIM/SULFAMETHOXAZOLE |
| 5 | CEFOTAXIME, CEFOTETAN, CEFOXITIN, CEFTRIAXONE, COLISTIMETHATE, DAPTOMYCIN, DOXYCYCLINE, GENTAMICIN, MINOCYCLINE, TELAVANCIN, TETRACYCLINE, TOBRAMYCIN, VANCOMYCIN |
| 6 | AMIKACIN, AMOXICILLIN-CLAVULANATE, AMPICILLIN-SULBACTAM, CEFEPIME, LINEZOLID, LINEZOLID, TEDIZOLID, TICARCILLIN-CLAVULANATE |
| 7 | AVIBACTAM/CEFTAZIDIME |
| 8 | CEFTAROLINE, CEFTOLOZANE/TAZOBACTAM, CIPROFLOXACIN, PIPERACILLIN-TAZOBACTAM |
| 9 | ERTAPENEM, LEVOFLOXACIN |
| 10 | DORIPENEM, MEROPENEM, MOXIFLOXACIN |
| 11 | IMIPENEM-CILASTATIN |
| 13 | TIGECYCLINE |

^a^based on the Antibiotic Spectrum Index developed by Gerber, J.S., *et al.,* [23].

**Figure S1.** Comparison of antibiotic use based on pharmacy invoices and dispensing data. To validate the dataset derived from pharmacy invoice data, we used dispensing data available from six nursing homes. Invoice data primarily represents individuals receiving skilled care; dispensing data include residents in skilled (short-stay) as well as those in residential/custodial (long-stay) care. Intravenous (IV) antibiotics are typically administered to residents receiving skilled care. Solid lines indicate total antibiotic use; dashed lines IV antibiotics. Light blue lines indicate dispensing data; orange lines indicate invoice data.


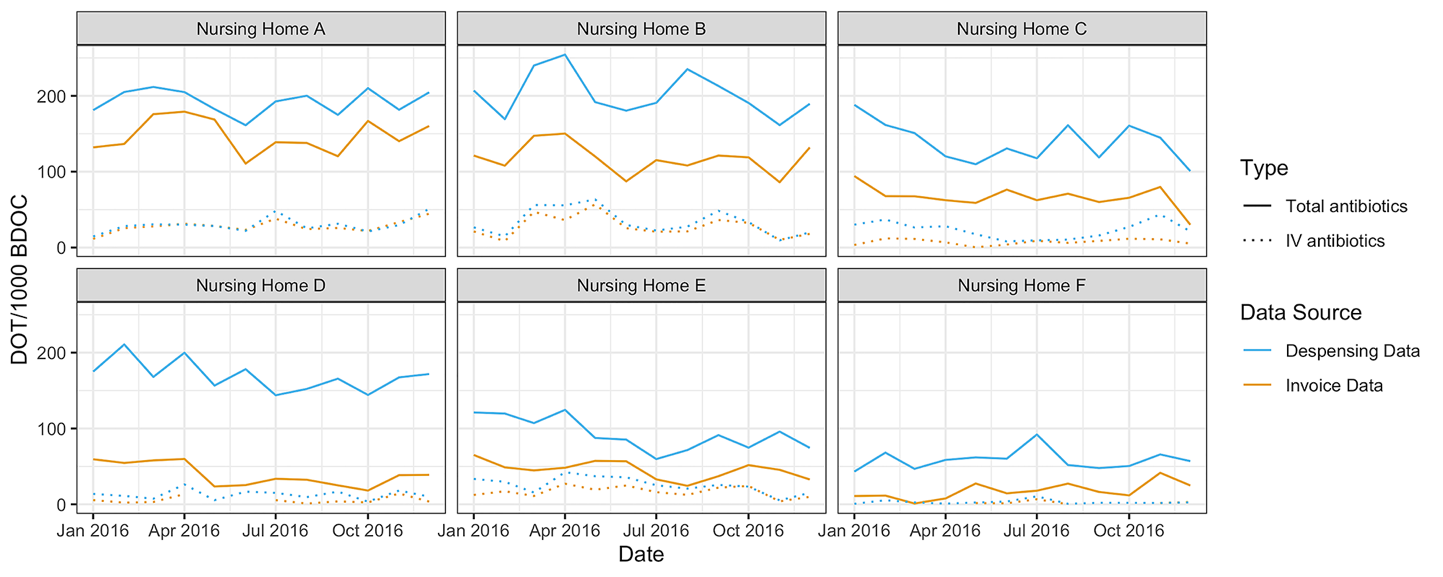


| **Nursing Home** | **% residents in skilled care** | **Number of Admissions/Bed**^a^ | **Correlation Rho-value (*P*-value) for All Antibiotics** | **Correlation Rho- value (*P*-value) for Intravenous Antibiotics** |
| --- | --- | --- | --- | --- |
| A | 32.84 | 4.73 | 0.69 (0.01) | 0.88 (<0.01) |
| B | 22.75 | 3.08 | 0.73 (0.01) | 0.99 (<0.01) |
| C | 23.62 | 1.76 | 0.81 (<0.01) | 0.39 (0.21) |
| D | 20.24 | 1.39 | 0.66 (0.02) | 0.81 (<0.01) |
| E | 27.77 | 1.04 | 0.51 (0.09) | 0.77 (<0.01) |
| F | 16.05 | 0.64 | 0.45 (0.14) | 0.60 (0.21) |

^a^Indicates the rate of resident turnover, with a higher number meaning a mean shorter length of stay, which is consistent with a higher proportion of residents receiving skilled nursing care.
